# VEXAS (Vacuoles, E1 Enzyme, X-linked, Autoinflammatory, Somatic) Syndrome in a Patient Presenting With Auricular Chondritis

**DOI:** 10.7759/cureus.111584

**Published:** 2026-06-26

**Authors:** Mildred Galvez, Anabella Pascucci

**Affiliations:** 1 Dermatology, David Geffen School of Medicine, University of California, Los Angeles, Los Angeles, USA

**Keywords:** autoinflammatory disease, chondritis, monoclonal gammopathy of undetermined significance, uba1 somatic mutation, vexas syndrome

## Abstract

VEXAS (vacuoles, E1 enzyme, X-linked, autoinflammatory, somatic) syndrome is a recently described autoinflammatory disorder caused by somatic UBA1 mutations in hematopoietic stem and progenitor cells. It typically presents in older males with treatment-refractory systemic inflammation and hematologic abnormalities. We report a case of a 92-year-old male who presented with auricular chondritis and other systemic symptoms, including inflammatory arthritis, conjunctivitis, and a diffuse rash. Laboratory evaluation revealed macrocytic anemia and elevated inflammatory markers, with largely negative autoimmune serologies aside from a mildly elevated rheumatoid factor. Bone marrow analysis showed cytoplasmic vacuolization of myeloid and erythroid precursors, and genetic testing identified a UBA1 p.Met41Thr pathogenic variant, confirming the diagnosis of VEXAS syndrome. Treatment with oral prednisone, monthly tocilizumab infusions, and supportive epoetin alfa-epbx led to significant clinical improvement. This case underscores the importance of considering VEXAS syndrome in elderly male patients presenting with unexplained systemic inflammation, chondritis, and cytopenias, and highlights the critical roles of early recognition, multidisciplinary evaluation, and timely genetic testing in diagnosis and management.

## Introduction

VEXAS (vacuoles, E1 enzyme, X-linked, autoinflammatory, somatic) syndrome is a recently characterized adult-onset autoinflammatory disorder, first described by Beck et al. in 2020 [1-3]. It involves both hyperinflammatory and hematologic dysfunction [1-3]. The defining diagnostic feature is a hematopoietic stem and progenitor cell-restricted somatic mutation in the UBA1 (ubiquitin-like modifier activating enzyme 1) gene, which encodes the E1 enzyme responsible for initiating the ubiquitin activation pathway [1-3]. Disruption of this pathway results in systemic inflammation and dysregulated hematopoiesis [2,3].

VEXAS syndrome occurs almost exclusively in males over the age of 50 years, consistent with the somatic and X-linked nature of the mutation [1-5]. Clinically, it can manifest with recurrent fevers, chondritis, arthritis, myalgias, ocular inflammation, pulmonary infiltrates, neutrophilic dermatoses, cutaneous vasculitis, venous thromboembolism, macrocytic anemia, and thrombocytopenia [1-5]. Many patients also present with treatment-refractory inflammation [1-3]. A characteristic bone marrow finding is cytoplasmic vacuolization of myeloid and erythroid precursors [1-3]. Diagnosis requires genetic confirmation of a UBA1 mutation, and management is often multidisciplinary [1-5].

We present a case of a 92-year-old male with auricular chondritis and macrocytic anemia, in the setting of previously diagnosed inflammatory arthritis and monoclonal gammopathy of undetermined significance (MGUS), who was subsequently diagnosed with VEXAS syndrome.

## Case presentation

A 92-year-old male presented to the outpatient dermatology clinic with several weeks of right ear erythema, edema, and pain. He had initially been evaluated at another clinic and prescribed amoxicillin-clavulanic acid for a presumed skin infection. Due to a lack of improvement, an otolaryngologist subsequently transitioned him to oral ciprofloxacin and topical mupirocin ointment, resulting in mild subjective improvement after one week. At the time of his dermatology evaluation, he also reported conjunctivitis, laryngitis, and sinusitis. His dermatologic history included actinic keratoses and non-melanoma skin cancer. Past medical history was notable for chronic kidney disease (CKD), nephrolithiasis, inflammatory arthritis managed by rheumatology, and MGUS under hematology-oncology evaluation.

**Figure 1:**
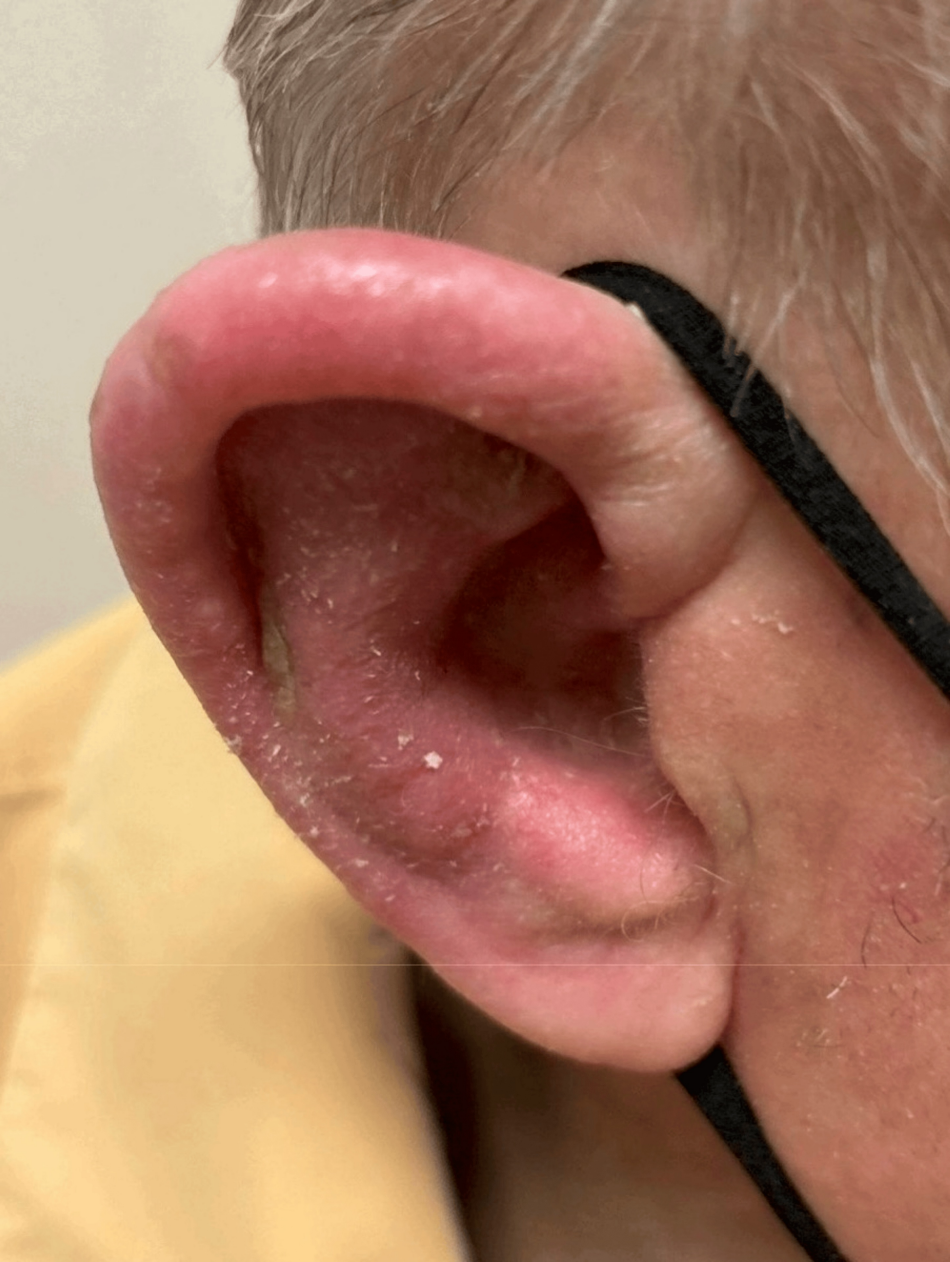
Auricular chondritis of the right ear at the time of initial evaluation. Erythema and edema of the right external ear with sparing of the earlobe.

Physical examination revealed tenderness, erythema, and edema of the right external ear with sparing of the earlobe (Figure 1). Laboratory studies from two weeks prior demonstrated macrocytic anemia with a normal white blood cell count (Table 1). Iron studies showed low serum iron, low total iron-binding capacity, and a markedly elevated ferritin. C-reactive protein and erythrocyte sedimentation rate were also elevated. Antinuclear antibody was within the reference range, while rheumatoid factor was mildly elevated. Vitamin B12 was elevated, and liver function tests were within normal limits.

**Table 1:** Key laboratory values at the time of initial evaluation. Abbreviations: WBC, white blood cell count; Hb, hemoglobin; Hct, hematocrit; MCV, mean corpuscular volume; TIBC, total iron-binding capacity; CRP, C-reactive protein; ESR, erythrocyte sedimentation rate; ANA, antinuclear antibody; RF, rheumatoid factor.

Lab test	Result	Reference range	Interpretation
WBC	8.08	4.16 - 9.95 (x10^3^/µL)	Normal
Hb	8.9	13.5 - 17.1 (g/dL)	Low
Hct	28.5	38.5 - 52 (%)	Low
MCV	101.8	79.3 - 98.6 (fL)	Elevated
Iron	28	41 - 179 (mcg/dL)	Low
TIBC	170	262 - 502 (mcg/dL)	Low
Ferritin	990	8 - 350 (ng/mL)	Elevated
Vitamin B12	1,951	254 - 1,060 (pg/mL)	Elevated
CRP	16.5	<0.8 (mg/dL)	Elevated
ESR	68	≤12 (mm/hr)	Elevated
ANA	<1:40	<1:40 (titer)	Normal
RF	19	<14 (IU/mL)	Elevated

The patient was started on triamcinolone 0.1% ointment and instructed to complete his ciprofloxacin course and continue using topical mupirocin. A biopsy was recommended but deferred per the patient’s request. Given the patient’s auricular chondritis and accompanying systemic inflammatory symptoms, the differential diagnosis included infection, relapsing polychondritis, and VEXAS syndrome. Return precautions were discussed, and the patient was advised to continue follow-up with rheumatology for further evaluation and to maintain ongoing hematology-oncology monitoring for MGUS.

**Figure 2:**
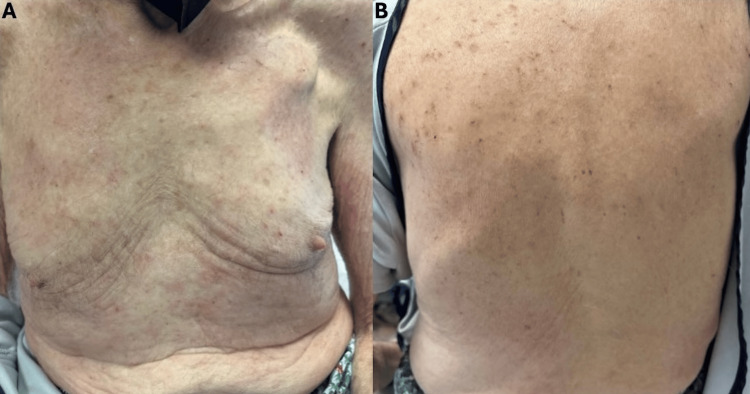
Truncal cutaneous eruption at the time of initial evaluation. Diffuse erythematous papules and macules on the chest and abdomen (A) and on the back (B), with a few excoriated lesions.

At his rheumatology visit one week later, he reported ongoing auricular tenderness; a pruritic, erythematous maculopapular rash on the torso (Figure 2) and bilateral upper and lower extremities that had been present for several months; and unintentional weight loss of approximately 4.5 kg over two months. Additional autoimmune serologies and computed tomography (CT) imaging were ordered to assess for infection, relapsing polychondritis, vasculitis, and occult malignancy. Genetic testing for a UBA1 mutation was also ordered, and the patient was referred to hematology-oncology for malignancy screening and evaluation for possible myelodysplastic syndrome (MDS). He was subsequently followed by rheumatology weekly, then monthly.

Laboratory testing for thyroid peroxidase antibodies, cyclic citrullinated peptide antibodies, and anti-neutrophil cytoplasmic antibodies (c-ANCA, p-ANCA) was negative. The patient’s auricular chondritis gradually improved with topical triamcinolone, oral ciprofloxacin, and topical mupirocin. Approximately one month after his initial dermatology visit, he was started on oral prednisone 30 mg daily with a gradual taper. Tuberculosis screening with interferon (IFN)-γ release assays was indeterminate, and hepatitis B and C serologies were negative. Prophylactic sulfamethoxazole-trimethoprim 800 mg-160 mg three times weekly and daily calcium-vitamin D supplementation were initiated. After one month of prednisone therapy, his arthritis, chondritis, and rash had improved.

Three months after his initial dermatology evaluation, CT angiography of the chest, abdomen, and pelvis demonstrated no evidence of infection, polychondritis, large vessel vasculitis, pulmonary embolism, or malignancy. Imaging also showed nonspecific lower lung-predominant ground-glass attenuation, favored to represent post-inflammatory changes, without associated clinical symptoms or need for further evaluation. Genetic testing identified the UBA1 c.122T>C mutation, corresponding to the p.Met41Thr variant. According to the pathology report, the bone marrow biopsy showed mild hypercellularity with multilineage hematopoiesis and no evidence of acute leukemia or a lymphoproliferative disorder, and the aspirate smear demonstrated cytoplasmic vacuoles within erythroid and myeloid precursors. Based on the genetic testing, bone marrow findings, and clinical presentation, a diagnosis of VEXAS syndrome was established.

The patient was advised to continue follow-up with rheumatology, hematology-oncology, and dermatology. After a discussion of therapeutic options, the patient elected to initiate monthly tocilizumab infusions (4 mg/kg) in combination with daily prednisone 15 mg, followed by a gradual taper. He was also started on epoetin alfa-epbx 20,000 units subcutaneously to target a hemoglobin level greater than 10 g/dL, given his anemia and CKD. At the two-month follow-up visit, clinical documentation indicated significant improvement, with near resolution of his arthritis, chondritis, rash, and other symptoms.

## Discussion

This case is notable for the diagnosis of VEXAS syndrome in a male at age 92, highlighting that recognition may be delayed in a recently defined syndrome with nonspecific, overlapping clinical features. Furthermore, a dermatologic presentation, specifically auricular chondritis, prompted further diagnostic evaluation and ultimately led to the diagnosis. His presentation, including chondritis, conjunctivitis, laryngitis, sinusitis, diffuse rash, weight loss, macrocytic anemia, and previously diagnosed inflammatory arthritis and MGUS, mirrored findings reported in other cases of VEXAS syndrome [1-6]. Laboratory abnormalities, including elevated inflammatory markers, markedly increased ferritin, elevated vitamin B12, and largely negative autoimmune serologies, were likewise consistent with previously described presentations [1,4,5]. His low serum iron and low total iron-binding capacity in the setting of elevated ferritin were also comparable to laboratory patterns reported in other patients with VEXAS syndrome [5,7].

Given the nonspecific and broad symptom profile, the initial differential diagnosis included infection, relapsing polychondritis, MDS, malignancy, and VEXAS syndrome. Diagnosis was established following detection of a pathogenic UBA1 p.Met41Thr variant, with further confirmation provided by the identification of cytoplasmic vacuoles in myeloid and erythroid precursors on bone marrow analysis. Prior to diagnosis, the patient had shown only a partial response to oral prednisone. After confirmation, he was treated with monthly tocilizumab infusions and a prednisone taper, along with epoetin alfa-epbx for anemia management, resulting in marked clinical improvement.

VEXAS syndrome predominantly affects males over the age of 50 and commonly presents with macrocytic anemia, elevated inflammatory markers, chondritis, cutaneous manifestations, and persistent inflammation refractory to treatment [1-6]. Diagnosis requires a mutation in the X-linked UBA1 gene in hematopoietic stem and progenitor cells, most often in myeloid cells, along with supportive clinical evidence [1]. Macrocytic anemia is a hallmark of the disease, with some patients also demonstrating iron studies consistent with anemia of inflammation, including low serum iron, low total iron-binding capacity, and elevated ferritin [5,7,8]. Recent work suggests that VEXAS syndrome-associated anemia may involve mosaic erythroblastopenia [8]. Macrocytosis may reflect wild-type UBA1 clonal hematopoiesis, erythroblast maturation arrest, or stress erythropoiesis, while chronic inflammation may promote clonal selection and create an unfavorable bone marrow environment [8]. In this setting, anemia is likely multifactorial, reflecting both inflammatory and hematologic mechanisms [8]. Among patients with VEXAS syndrome, the clinical severity of anemia is variable, with some patients developing transfusion dependence [2,7].

Many patients also have concomitant hematologic conditions, such as MDS, MGUS, and plasma cell myeloma [1-3]. UBA1 mutations have been associated with an increased predisposition to these disorders [3]. Given the somatic nature of these mutations, clonal expansion over time may contribute to the adult-onset presentation characteristic of VEXAS syndrome [3]. Furthermore, the location of the gene on the X chromosome contributes to the increased prevalence in males compared to females, who have an additional, unaffected copy of the UBA1 gene [1,2,9]. The UBA1 p.Met41Thr variant found in this patient is the most frequently reported pathogenic variant, followed by p.Met41Val and p.Met41Leu [1,2,10]. However, novel variants continue to be identified, expanding the clinical and genetic spectrum of this syndrome [2,7,11].

UBA1 encodes the E1 enzyme responsible for initiating the ubiquitin activation pathway, which plays a central role in protein and cellular homeostasis, cell cycle control, apoptosis, and immune signaling [2-5]. Disruption of this pathway can lead to systemic inflammation and dysregulated hematopoiesis and has also been implicated in other disorders, including neurodegenerative diseases and malignancies [2-5]. Disease-causing UBA1 mutations have been described in VEXAS syndrome and X-linked spinal muscular atrophy, a congenital neuromuscular disease [9]. Somatic UBA1 variants have also been reported in association with certain hematologic malignancies and lung cancer [9].

Patients with VEXAS syndrome have highly activated inflammatory signatures across multiple pathways, including those involving tumor necrosis factor (TNF), interleukin (IL)-6, and IFN-γ, which have informed emerging treatment strategies [1,5]. Current therapeutic approaches focus on immunomodulation and targeting UBA1-mutated clones. Treatment options include corticosteroids and Janus kinase (JAK) inhibitors (ruxolitinib and tofacitinib) [2-5,11]. Cytokine-directed therapies have also been used and include anti-TNF-α agents (adalimumab), IL-6 receptor blockers (tocilizumab), and anti-IL-1 therapies (anakinra and canakinumab) [2-5,11]. Other agents include methotrexate, calcineurin inhibitors (cyclosporine), and hypomethylating agents (azacitidine) [2-5,11]. Currently, treatment recommendations remain largely guided by observational evidence rather than prospective clinical trials [2,12]. Glucocorticoids remain the most effective option for managing the inflammatory symptoms associated with VEXAS syndrome [1,12]. Although immunomodulatory therapy remains the primary management approach, allogeneic hematopoietic stem cell transplantation may offer a potentially curative option [1,2,9,11]; however, its use is limited by the associated risks and lack of long-term outcome data [2,9].

VEXAS syndrome carries substantial morbidity and mortality, often due to progressive anemia, coexisting hematologic disorders, disease-associated respiratory failure, or treatment-related adverse events [1-3]. This case highlights the importance of recognizing characteristic clinical features, confirming the diagnosis through genetic testing, and implementing multidisciplinary management.

## Conclusions

This case underscores the importance of identifying clinical patterns suggestive of VEXAS syndrome in older male patients presenting with treatment-refractory systemic inflammation, chondritis, or other characteristic features, and cytopenias. Early recognition is critical, as diagnosis requires genetic confirmation of a UBA1 mutation supported by cytopathologic evidence of cytoplasmic vacuolization within myeloid and erythroid precursor cells. Timely identification can help guide appropriate immunomodulatory therapy, reduce unnecessary treatments, and help prevent disease-related complications. Given its multisystem involvement and heterogeneous presentation, optimal management requires a multidisciplinary approach involving dermatology, rheumatology, and hematology-oncology. As awareness of this recently defined syndrome increases, continued case reporting and research will further expand understanding of its clinical variability, genetic spectrum, and therapeutic responses, ultimately improving patient care and outcomes.
